# Fusion Attention for Action Recognition: Integrating Sparse-Dense and Global Attention for Video Action Recognition

**DOI:** 10.3390/s24216842

**Published:** 2024-10-24

**Authors:** Hyun-Woo Kim, Yong-Suk Choi

**Affiliations:** 1 Department of Artificial Intelligence Application, Hanyang University, Seoul 04763, Republic of Korea; commiya2@hanyang.ac.kr; 2 Department of Computer Science and Engineering, Hanyang University, Seoul 04763, Republic of Korea

**Keywords:** action recognition, fusion attention, temporal redundancy

## Abstract

Conventional approaches to video action recognition perform global attention over the entire video patches, which may be ineffective due to the temporal redundancy of video frames. Recent works on masked video modeling adopt a high-ratio tube masking and reconstruction strategy as a pre-training method to mitigate the problem of focusing on spatial features well but not on temporal features. Inspired by this pre-training method, we propose Fusion Attention for Action Recognition (FAR), which fuses the sparse-dense attention patterns specialized for temporal features with global attention during fine-tuning. FAR has three main components: head-split sparse-dense attention (HSDA), token–group interaction, and group-averaged classifier. First, HSDA splits the head of multi-head self-attention to fuse global and sparse-dense attention. The sparse-dense attention is divided into groups of tube-shaped patches to focus on temporal features. Second, token–group interaction is used to improve information exchange between divided patch groups. Finally, the group-averaged classifier uses spatio-temporal features from different patch groups to improve performance. The proposed method uses the weight parameters that are pre-trained with VideoMAE and MVD, and achieves higher performance (+0.1–0.4%) with less computation than models fine-tuned with global attention on Something-Something V2 and Kinetics-400. Moreover, qualitative comparisons show that FAR captures temporal features quite well in highly redundant video frames. The FAR approach demonstrates improved action recognition with efficient computation, and exploring its adaptability across different pre-training methods presents an interesting direction for future research.

## 1. Introduction

In recent years, the self-attention-based transformer [1] architecture has achieved impressive performance in natural language processing [2,3,4]. Vision transformers [5] have also been proposed in computer vision, achieving state-of-the-art performance in tasks such as classification [6], object detection [7], segmentation [8], and video recognition [9,10,11]. Linearly projected image/video tokens can use multi-head self-attention to model global dependencies between spatial and temporal visual content. The attention mechanism effectively minimizes inductive bias.

Video action recognition has become an important task in computer vision, impacting areas such as autonomous driving and human–computer interaction. Traditional methods for action recognition typically rely on CNN-based architectures [12,13,14], including 3D convolutional networks and two-stream networks, which have achieved considerable success. However, these models often face limitations related to their inherently limited receptive fields, which hinder their ability to effectively capture long-range spatio-temporal dependencies. In recent years, transformer-based approaches, in particular, vision transformers [10,11,15,16,17], have demonstrated state-of-the-art performance by using multi-head self-attention to model both spatial and temporal dependencies simultaneously. Despite these advances, many existing methods suffer from focusing on spatial features while underutilizing temporal information due to the inherent temporal redundancy of video data.

**Figure 1:**
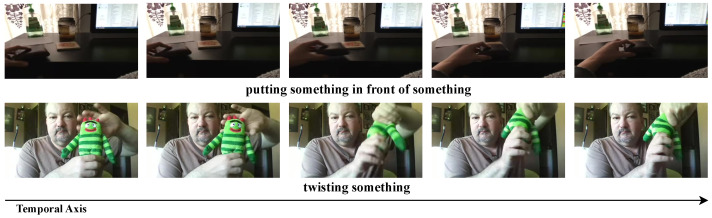
Two classes in the Something-Something V2 dataset. Spatial features are slower to change over time, and there are more spatial features than temporal features [18].

The video has two characteristics: temporal redundancy [18] and correlation. The spatial features of each frame change slowly over time, resulting in adjacent frames being highly redundant, as shown in Figure 1. This temporal redundancy causes the model to learn by focusing on the spatial information rather than the scarce temporal information. Video also has an inherent correlation between adjacent frames. In self-supervised video representation learning, the masked autoencoder [9,19,20] uses an asymmetric encoder-decoder architecture to achieve state-of-the-art performance, where high-ratio tube masking is performed by considering temporal redundancy and correlation to obtain pre-training weights that are good at capturing scarce temporal features of the video data. The simple strategy of using vanilla ViT [5] means that impressive reconstruction results can be achieved and masked patches can be complemented with limited visual content. This is because even if a high percentage of patches are discarded, the temporal redundancy inherent in video allows the model to recover visual information from parts of the video that were not seen during the pre-training phase. After pre-training, patches from all videos are typically fed into the model to fine-tune it in a downstream action recognition task.

In this study, we argue that use of global attention only across all patches during fine-tuning in a downstream action recognition task is not an effective method given video characteristics. In the pre-training phase, the masking strategy [9,19] considers temporal redundancy and correlation but not during fine-tuning, leading to a focus on rich spatial features. To address this, we propose a new method for the action recognition task called Fusion Attention for Action Recognition (FAR). The key idea of FAR is to use explicit sparse-dense attention patterns, which effectively model temporal features and fuse them with existing global attention.

**Figure 2:**
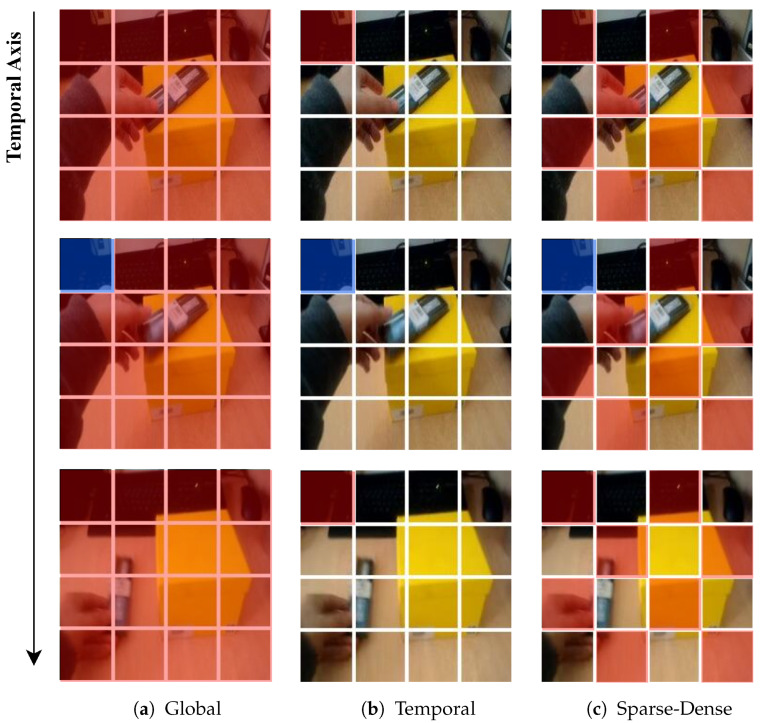
Visualization of the three attention schemes used in this study. Each patch has 2 × 16 × 16 of pixel information. Query patches are colored blue, and patches without color are not used in the self-attention computation. Self-attention is computed for every single patch in the video clip, with every patch serving as a query. The attention pattern is only shown for two adjacent frames, but it extends to all frames in the clip in the same way.

The existing VideoMAE [19] and MVD [9] consider the temporal redundancy [18] and correlation of videos and adopt a high rate (i.e., 90% to 95%) of tube masking to prevent “information leakage”, which increases the reconstruction difficulty. In contrast to the pre-training strategy, the fine-tuning phase does not consider the characteristics of the video and only applies global attention to all video patches, as shown in Figure 2a. This approach leads to the problem that the model is trained by focusing on the video’s rich spatial features and is poorly able to capture the temporal features. To solve this problem, we designed an explicit attention pattern that divides the video patch into dense along the temporal axis but sparse along the spatial axis, as shown in Figure 2c. More specifically, unlike global attention, sparse-dense attention models temporal features by dividing the video patch into groups in the form of tubes that are dense along the temporal axis but sparse along the spatial axis to form a pattern. Then, to fuse with global attention and apply it to the model, the head of the MHSA is divided into specific proportions to model different features of the two attention schemes.

Nevertheless, we observed that the performance is similar to or slightly lower than the baseline model [9,19] using only global attention. Although temporal features can be obtained more effectively, there is a problem that the exchange of feature information between different video patch groups is insufficient because attention is divided into two groups. To solve this problem, we added a token–group interaction module with a bottleneck convolution structure to the attention block to exchange information between groups to effectively integrate spatio-temporal features. In a typical ViT [5], all visual patches are averaged into one token and fed to the FC layer. In sparse-dense attention, the patches are divided into two groups, and the two groups capture different spatio-temporal features. However, when a single token is used by averaging all visual patches in the last FC layer, the features obtained are diluted. Therefore, we propose a group-averaged classifier that uses two tokens by averaging over patches in the same group of sparse-dense attention patterns.

We show that this method achieves better performance compared to existing downstream action recognition approaches [9,19]. It mitigates temporal redundancy, captures temporal features better than existing methods, and effectively integrates them into spatio-temporal features. The contributions of this research can be summarized as follows:

- For effective video action recognition, we propose a new FAR consisting of head-split sparse-dense attention, token–group interaction, and group-averaged classifier to mitigate temporal redundancy and effectively capture spatio-temporal features.
- On the Something-Something V2 [21], Kinetics-400 [22] dataset, the FAR outperforms a model using only global attention trained with the same pre-training method by 0.1–0.4% with slightly lower computational cost.

## 2. Related Work

Action recognition is a challenging and important problem in vision, and it is a fundamental task for video analysis and understanding. Recently, video networks have been categorized into CNN-based and Transformer-based.

**CNN-based methods.** CNN-based methods are typically built as 3D convolutional networks [12,13,14]. C3D [12] trains a VGG network with a 3D CNN to learn the spatio-temporal features of a video. I3D [13] extends the weights of an Inception [23] model pre-trained with ImageNet from a 2D CNN to a 3D CNN. SlowFast [14] uses 2-stream 3D convolution to model spatial appearance and temporal relationship separately at different frame rates. However, 3D convolution suffers from excessive computational complexity. P3D [24] factorizes the 3D CNN into a spatial (2D) CNN and a temporal (1D) CNN to reduce the computational complexity. Other works use 2D convolution for spatial modeling and then introduce additional temporal operations, such as weight calibration [25,26], adaptive temporal kernel [27], and motion modeling [28,29,30], for temporal feature modeling. Convolution-based methods struggle to model global spatio-temporal dependencies within the same image or between different images due to small receptive fields.

**Transformer-based methods.** In recent years, transformer-based networks have made significant progress in the image domain and have been applied to video networks [10,11,15,16,17]. To achieve superior performance compared to CNN-based architectures, some works adapt vision transformers to the video domain. For example, TimeSformer [15] and ViViT [11] adopt spatio-temporal factorization methods to extend vision transformers to the video domain. MViT [17] and VideoSwin [10] adopt hierarchical architectures, introduce inductive bias, and achieve a speed-accuracy trade-off. The uniformer [16] efficiently integrates 3D CNN and spatio-temporal self-attention. In this paper, we adopt vanilla ViT weights pre-trained with VideoMAE [19] and MVD [9]. We show that FAR can be used to fine-tune the pre-trained vanilla ViT weights effectively.

**Temporal redundancy.** Unlike static images, video involves a temporal relationship. However, semantics change slowly along the temporal axis [18], and models are trained by focusing on rich spatial features rather than capturing scarce temporal features. Reducing temporal redundancy is a popular research topic for effective video analysis. Adaptive frame selection [31,32,33] dynamically determines which frames have important temporal features using an additional lightweight network. MGSampler [34] uses distributions to select frames that contain motion information. ILA [35] reduces temporal redundancy with learnable alignment and achieves competitive performance with explicit temporal modeling. VideoMAEV2 [36] reduces temporal redundancy through dual masking and proposes a billion-parameter model. Our FAR uses an explicit attention pattern that divides patches into two groups, rather than sampling specific frames, to effectively capture the temporal features that are lacking during fine-tuning.

**Masked autoencoder self-supervised video pre-training (SSVP).** Masked language modeling [2], which learns effective representations by reconstructing corrupted input, has been one of the main pre-training methods in the NLP domain. Masked visual modeling was introduced to self-supervised visual pre-training due to the success of the vision transformer and has been shown to be useful for multimodal visual-language learning [37,38]. BEiT [39], PeCo [40], and VIMPAC [41] followed BERT [2] and proposed to learn visual representations of images and videos by predicting discrete tokens pre-trained by VQ-VAE. MAE [42] introduces an asymmetric encoder-decoder framework for masked pixel reconstruction, which significantly reduces the computational cost of image modeling. SimMIM [43] and MaskFeat [44] have been designed to recover low-level features, such as pixels and HOGs. Hiera [45] achieves high accuracy in video classification by applying MAE pre-training to a vision transformer with a hierarchical structure. VideoMAE [19] and ST-MAE [20] follow the asymmetric encoder-decoder structure of MAE [42] and mask a high percentage of the video patch. Unlike ST-MAE [20], which performs random masking, VideoMAE [19] performs high ratio tube masking by considering temporal redundancy and correlation. MVD [9] follows the tube masking strategy by pre-training ImageMAE [42] and VideoMAE [19] and then using them as teacher models to help the student model learn the high-level features of images and videos. The goal of the masked autoencoder SSVP is to be able to learn the missing temporal features, and our FAR uses the pre-training weights of VideoMAE [19] and MVD [9] to capture the lacking temporal features during fine-tuning.

## 3. Methodology

In this section, we present the proposed Fusion Attention for Action Recognition (FAR), which utilizes ViT pre-trained with a masked autoencoder [9,19] to mitigate temporal redundancy [18] and effectively perform the downstream video action recognition task. In the following, we first describe how to build a video-based vision transformer. Then, we introduce the components of the proposed FAR: head-split sparse-dense attention, which fuses two attention patterns to obtain good temporal information of the video, and token–group interaction and group-averaged classifier design, which integrate the obtained spatio-temporal features.

### 3.1. Video Vision Transformer

The video vision transformer is extended based on the image ViT [5]. The input video clip $X\in R^{F\times H\times W\times C}$ is divided into non-overlapping s×k×k patches. The tube-shaped 3D patches are flattened by $x^{\left(t,p\right)}\in R^{3sk^{2}}$, where t=1,…,T denotes the temporal index (T=F/s) and p=1,…,N denotes the spatial index $\left(N=HW/k^{2}\right)$. The video patches are then mapped to tokens using a linear layer.

(1) $$\begin{array}{c} {z_{0}}^{\left(t,p\right)}=Ex^{\left(t,p\right)}+e_{\left(pos\right)}^{\left(t,p\right)} \end{array}$$

where ${z_{0}}^{\left(t,p\right)}$ is the video patch at position (t,p), $E\in R^{3sk^{2}\times D}$ is the weight of the linear layer, and $e_{\left(pos\right)}^{\left(t,p\right)}$ is the sin-cos positional embedding.

The entire sequence of a video patch is denoted as $Z_{0}$, and it is assumed that ViT has *L* encoders, each consisting of Multi-Head Self-Attention (MHSA), Layer Norm (LN), and FFN. ViT encoders can be represented as follows:

(2) $$\begin{array}{c} \begin{array}{cc} \; & Z_{l}^{\prime }=\mathrm{MHSA}\left(\mathrm{LN}\left(Z_{l-1}\right)\right)+Z_{l-1},\\ \; & Z_{l}=\mathrm{FFN}\left(\mathrm{LN}\left(Z_{l}^{\prime }\right)\right)+Z_{l}^{\prime }, \end{array} \end{array}$$

where $Z_{l}^{\prime }$ and $Z_{l}$ denote the output features of the MHSA and FFN for encoder block *l*. The MHSA is computed as follows (LN is omitted for convenience):

(3) $$\begin{array}{c} \begin{array}{cc} \; & \mathrm{Attention}\left(Q,K,V\right)=\mathrm{SoftMax}\left(QK^{T}/\sqrt{d}\right)V,\\ \; & \mathrm{MultiHead}\left(Q,K,V\right)=\left[head_{1},\ldots ,head_{h}\right]\cdot W^{O}\\ \; & head_{i}=\mathrm{Attention}\left(Z_{l-1}^{i}W_{i}^{Q},Z_{l-1}^{i}W_{i}^{K},Z_{l-1}^{i}W_{i}^{V}\right), \end{array} \end{array}$$

where Q,K,V are the query, key, and value used in the attention computation. *d* is the scaling factor equal to the dimension of query,key. $W_{i}^{Q}$, $W_{i}^{K}$, and $W_{i}^{V}$ are the linear layers for the mapping, respectively, and i=1,…,h denotes the head index of the MHSA. The MHSA is computed by dividing the embedding dimension by the number of heads.

Typically, the token fed to the classifier is averaged over all video patches. The $Z_{L}$ from the last encoder layer is fed to the classifier as follows:

$$\mathrm{Action}\;\mathrm{Class}=\mathrm{FC}\;\mathrm{Layer}(\mathrm{GlobalAverage}\left(Z_{L}\right))$$

ViT performs only global attention, where each head computes an attention score for every visual patch. Our FAR splits the heads in two to perform global attention and sparse-dense attention simultaneously to focus more on temporal features and uses token–group interaction and group-averaged classifiers to capture spatio-temporal features.

### 3.2. Proposed Method

**Discussion of attention pattern.** The pre-training, which utilizes the characteristics of the video data, performs high-ratio tube masking [9,19] (i.e., 90% to 95%) to obtain weights that effectively capture the scarce temporal information. However, during fine-tuning, only global attention is applied to all patches, as shown in Figure 2a. This approach focuses on the rich spatial features rather than the scarce temporal features due to the temporal redundancy of video. Therefore, in order to overcome this during fine-tuning, we investigate types of attention that focus more on temporal features.

Temporal attention is a scheme proposed by TimeSformer [15]. Unlike global attention, it divides video patches into groups, as shown in Figure 2b. Specifically, patches at the same spatial location in different frames are sampled in a tube shape to belong to the same attention group. Temporal attention can be represented as follows:

(4) $$\begin{array}{cc} \; & \mathrm{TemporalAttention}\left(Q,K,V\right)=\mathrm{SoftMax}\left(Q_{p}K_{p}^{T}/\sqrt{d}\right)V_{p} \end{array}$$

where p=1,…,N denotes the group index, and *N* denotes the number of patches in one frame. The tube-shaped groups of patches are dense along the temporal axis, but no attention is paid to patches in the same frame. This pattern can model temporal features. However, it learns very few spatial features for fine-tuning downstream tasks, and the model is too hard to train, resulting in poor performance.

Therefore, we propose sparse-dense attention, which is an extension of temporal attention. After sampling in the same way as temporal attention, the tube patch groups are divided into two groups in a checkerboard pattern as shown in Figure 2c. This way of sampling allows us to organize the groups densely along the temporal axis and sparsely along the spatial axis. Sparse-dense attention can be represented as follows:

(5) $$\begin{array}{cc} \; & \mathrm{Sparse}-\mathrm{Dense}\;\mathrm{Attention}\left(Q,K,V\right)=\mathrm{SoftMax}\left(Q_{g}K_{g}^{T}/\sqrt{d}\right)V_{g} \end{array}$$

where g=1,2 denotes the group index. In contrast to temporal attention, patches in the same frame are also grouped together, which can lead to better performance in fine-tuning downstream tasks. However, changing the attention pattern of each block to sparse-dense results in a lack of spatial features. To address this issue, we propose head-split sparse-dense attention (HSDA), which balances temporal and spatial features by fusing global and sparse-dense attention.

**Figure 3:**
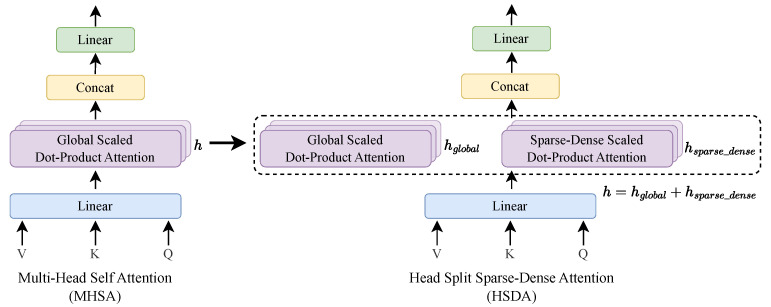
Structure of the proposed HSDA. Split the head of MHSA and perform global attention and sparse-dense attention computation in the head, respectively. MHSA and HSDA have the same total number of heads.

**Head-split sparse-dense attention.** The method of our proposed Head-Split Sparse-Dense Attention (HSDA) is shown in Figure 3. The HSDA is computed as follows:

(6) $$\begin{array}{c} \begin{array}{c} \mathrm{HSDA}\left(Q,K,V\right)=\left[\mathrm{GlobalHead};\mathrm{SparseDenseHead}\right]\cdot W^{O} \end{array} \end{array}$$

The heads in the MHSA are split according to a specific ratio, dividing them into GlobalHead, which performs global attention, and SparseDenseHead, which performs sparse-dense attention. In each head, the GlobalHead and SparseDenseHead are combined after computing according to the attention pattern. We found that HSDA, which fuses the two attentions in a single block, requires about 10% less computation than using global attention alone, with little loss in performance.

**Token–group interaction.** HSDA fuses sparse-dense and global attention to better capture temporal features. However, the ability to capture spatio-temporal features becomes insufficient due to the lack of information exchange between different groups of video patches. To address this issue, we added a token–group interaction in the bottleneck group convolution structure to exchange information between different video patch groups before the block’s attention. The convolution group is equal to the number of heads in HSDA, which ensures effective and efficient information exchange between channel features belonging to the same head. By adding this module, information from different patch groups can be modeled before attention, and spatio-temporal features can be effectively integrated.

**Figure 4:**
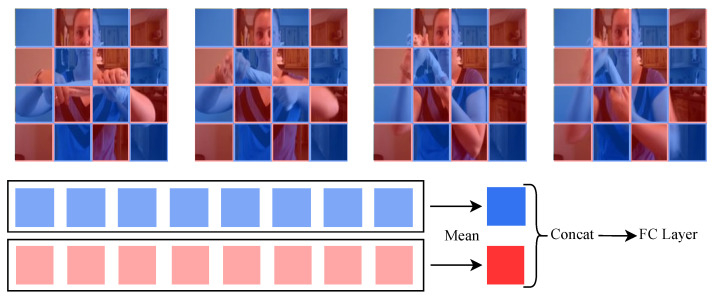
Structure of the proposed group-averaged classifier. The video patches are divided into two groups, red and blue, based on sparse-dense attention patterns. The average operation is performed between patches belonging to the same group, and the two tokens are combined and fed to the FC layer.

**Group-averaged classifier.** A commonly used classifier generates a single token at the end by averaging over all patches and feeds it to the FC layer. This approach works well for general ViTs with only global attention, but with sparse-dense attention, the features obtained are diluted when combined into a single token. Our group-averaged classifier was designed to overcome this problem and improve performance. In sparse-dense attention, the patches are divided into two groups, and the two groups capture different spatio-temporal features. Therefore, instead of averaging over all video patches at once to generate a single token, we average over patches belonging to the same group to obtain two tokens that contain the spatio-temporal features of each group, as shown in Figure 4. The two tokens are then combined into one and fed to the FC layer. Constructing a classifier using the average token from each group adds very few parameters and little computation but effectively increases performance.

### 3.3. Fusion Attention for Action Recognition (FAR)

**Figure 5:**
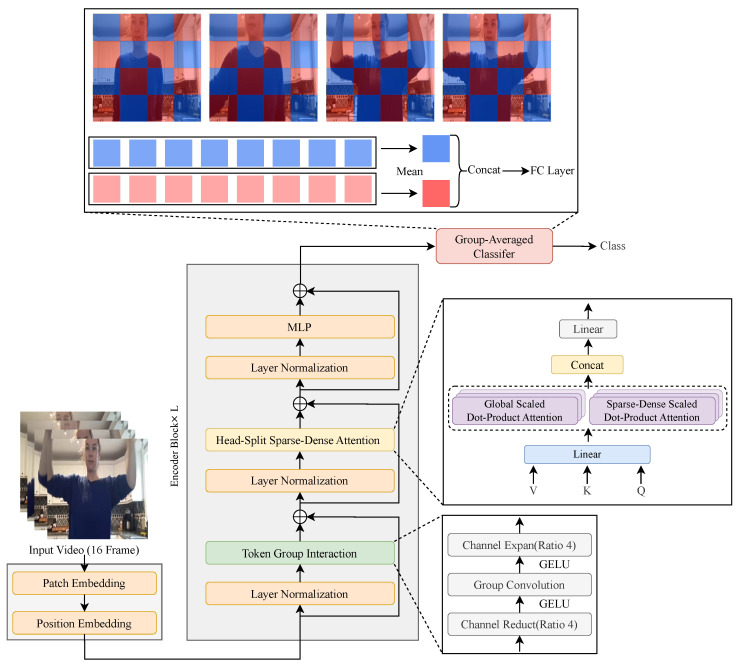
Overview of FAR building blocks.

Based on the proposed HSDA, token–group interaction, and group-averaged classifier, we build FAR, which captures effective spatiotemporal features, as shown in Figure 5. In each block, the MHSA of ViT [5] is replaced by HSDA, the token–group interaction is added, and the last classifier is replaced by the group-averaged classifier. FAR can use powerful weights pre-trained with masked autoencoders [9,19]. FAR’s token–group interaction and classifier have random initialization, while the rest of the modules use existing pre-trained weights.

## 4. Experiments

### 4.1. Datasets

We train and evaluate our model on two downstream video action recognition tasks, utilizing weights from an existing pre-trained [9,19] vanilla ViT. Each dataset has unique characteristics for understanding action recognition in different contexts.

Something-Something V2 (SSv2) [21] contains 174 classes focused on object interactions, with 169 k training videos and 20 k validation videos. The average video length is 4 s, emphasizing subtle, action-oriented gestures, such as pushing, pulling, or twisting objects. A key challenge with SSv2 is its high temporal redundancy; small spatial variations over time require the model to effectively capture fine-grained temporal features to distinguish between similar actions.

Kinetics-400 (K400) [22] contains 400 human motion classes covering a broad range of activities, such as sports and everyday life. Each video is 10 s long on average, with a minimum of 400 examples per class, resulting in 240,000 training videos and 20,000 validation videos. Unlike SSv2, K400 includes more complex spatio-temporal patterns and varied backgrounds, requiring the model to handle different contexts and effectively capture both global and local motion patterns. These two datasets allow the model to validate its performance across a variety of video contexts, from fine-grained object manipulation to broad, complex human behaviors.

### 4.2. Implementation Details

MVD [9] and VideoMAE [19] are selected as pre-trained models with masked autoencoders, and FAR-Small, FAR-Base models are developed based on ViT-Small, Base. To conveniently use the pre-trained models, we use the same spatio-temporal patches as VideoMAE and MVD (i.e., 2×16×16). The spatio-temporal resolution of the input video is 16×224×224 for both training and inference. For reproducibility, we detail the complete experimental setup. Fine-tuning experiments are conducted on four NVIDIA A5000 GPUs using the SSv2 and K400 datasets, with DeepSpeed employed for memory efficiency and fast training. The hyperparameters, such as optimizer settings (AdamW), learning rate, weight decay, batch sizes, and data augmentation strategies (e.g., Mixup, CutMix, and RandAugment), are optimized for both datasets and are found in Table 1.

### 4.3. Ablation Study

In this subsection, we describe an ablation study on FAR using Something-Something V2 [21]. We use ViT-S pre-trained with MVD [9] for performance and efficiency as the default backbone and take 16 frames as input. All ablation experiments use the same inference and training settings.

**Attention pattern.** Changing the attention pattern is one of the key components of FAR. In Table 2, we have experimented with different attention patterns. The first compartment uses a single pattern, and the second compartment is the result of experiments with the head-split scheme. Single-pattern experiments show that temporal attention has a significant performance drop, but sparse-dense attention does not. This shows that dividing the video patch into two groups due to the temporal redundancy of the video data does not result in significant performance degradation. The experimental results of the head-split method show that fusing temporal and global attention causes a significant performance drop, but using our proposed head-split sparse-dense attention, the computation is reduced by about 10%, but the performance drop is only 0.1%. Therefore, we set head-split sparse-dense as the default attention pattern.

**Token–group interaction.** Table 3 describes the effect of token–group interaction and the efficiency of group convolution. This ablation study aims to evaluate the impact of inter-group information exchange on the model’s overall performance in capturing spatio-temporal features. The token–group interaction module results in a performance improvement of 0.3%. This shows that this module effectively fuses spatio-temporal features by exchanging information between different patch groups. The performance difference between using group convolution in the middle of a bottleneck convolution and not using it is not significant. Setting the number of groups in the convolution to the same number of heads in the attention block allows for efficient and effective information exchange between channel features belonging to the same head. Therefore, we use group convolution to utilize a relatively small number of parameters and computations.

**Classifier type.** In Table 4, we compare the performance results of different classifiers. The purpose of this comparison is to determine the effectiveness of group-specific averaging in preserving distinct spatio-temporal information captured by each attention group. The group-averaged classifier achieves a 0.2% improvement over the global-averaged classifier. It also keeps the parameters and computation almost unchanged. These results show that the group-averaged classifier can effectively use the features of two patch groups to improve performance. Based on these experiments, we use a group-averaged classifier.

**Head-split ratio.** The global, sparse-dense head ratio is one of the key components. The experiment aims to determine the optimal balance between global and sparse-dense attention mechanisms to improve the capture of temporal and spatial features. We report experiments with different ratios in Table 5. In HSDA, increasing the number of global heads makes the model learn by focusing on spatial features. Conversely, increasing the number of sparse-dense heads makes the model learn by focusing on temporal features. The experiments show that a 1:1 ratio of heads results in improved performance, but a 2:1 or 1:2 ratio results in 0.4% lower performance. Having an equal number of heads, rather than focusing on a particular attention pattern, can balance spatial and temporal features. Therefore, we set the head-split ratio in HSDA to 1:1.

**Token–group interaction position.** We conducted experiments to determine the optimal location for incorporating the token–group interaction module into the FAR model, aiming to maximize the model’s effectiveness in capturing spatio-temporal features. We experimented with three different configurations, as shown in Table 6. When we placed the token–group interaction module before the attention layer, the model achieved the highest performance with a Top-1 accuracy of 71.1%. However, placing the token–group interaction module after the attention layer slightly decreased the Top-1 accuracy to 70.8%, and placing it after the MLP resulted in the lowest performance. These experimental findings indicate that better performance can be achieved by placing the token–group interaction module before the attention layer to effectively fuse spatio-temporal information.

### 4.4. Comparisons with Previous Methods

We compare our FAR with previous studies on two datasets of video action recognition tasks [21,22]. The tables include the relevant experimental settings, such as pre-training methods, model backbones, and computational requirements measured in FLOPs. The comparison involves three types of methods: supervised fine-tuning, masked autoencoder-based pre-training, and our proposed FAR approach.

The first compartment of each table includes methods based on supervised fine-tuning, such as SlowFast and MViTv2, which are popular for their high performance but have considerable computational overheads [10,11,14,15,17,51]. The second and third compartments highlight methods based on masked autoencoder self-supervised pre-training [20,41,44,52,53], which can be more computationally efficient while retaining competitive accuracy. The third compartment includes the pre-training method [9,19] that our FAR utilizes. VideoMAE and MVD are both fine-tuned using ViT, employing global attention exclusively. In contrast, our proposed FAR employs Fusion Attention, which combines sparse-dense attention with global attention, enabling the model to better balance the focus between spatial and temporal features.

The results for Something-Something V2 are shown in Table 7. It can be observed that methods based on masked autoencoder self-supervised video pre-training outperform supervised methods (see the first compartment of Table 7). Our FAR, based on the same pre-training method, achieves a 0.2% increase in accuracy compared to VideoMAE, with slightly less computational cost. FAR also outperforms MVD by 0.4% and 0.1% in different configurations, consistently demonstrating competitive performance at a lower computational cost. These results indicate that FAR can effectively model temporal features while being more efficient in terms of FLOPs.

The results for Kinetics-400 are shown in Table 8. The results for FAR also show an increase in accuracy compared to both VideoMAE and MVD. Specifically, they show an improvement of 0.4% over VideoMAE, while outperforming MVD by 0.2%. These results validate the effectiveness of our HSDA mechanism in capturing spatio-temporal dynamics that are critical for video action recognition tasks. These improvements demonstrate that FAR can better capture the temporal relationships that global attention often fails to capture in cases of significant temporal redundancy.

### 4.5. Qualitative Comparison

To validate that FAR was designed as intended, we conducted visualizations of MVD and FAR in Something-Something V2, and of VideoMAE and FAR in Kinetics-400. Specifically, we used GradCAM [54] to compare the results to see which regions the model focuses on during classification. In frames with high redundancy, the model focuses on regions with temporal features, which helps to improve classification performance.

**Figure 6:**
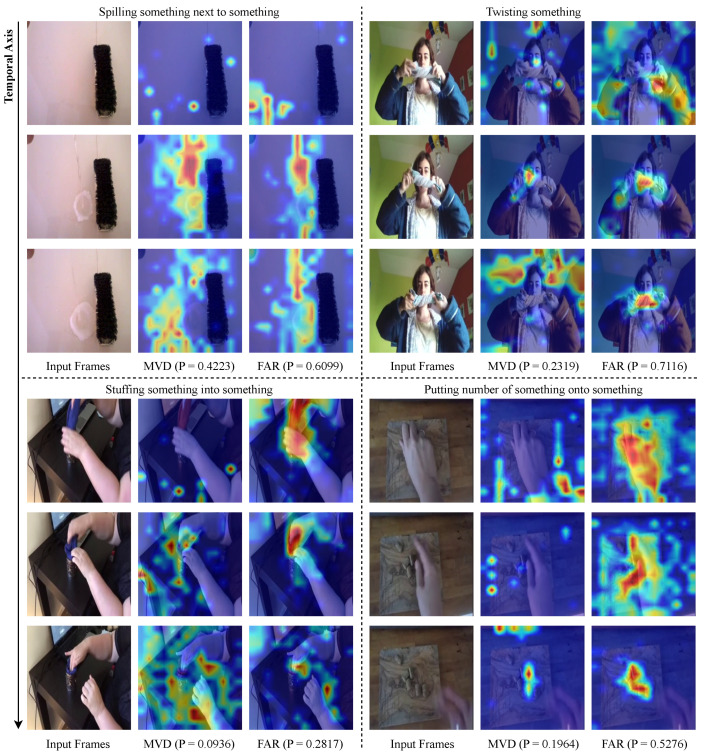
Qualitative comparison of MVD and FAR in Something-Something V2. Visualized with GradCAM, the heatmap shows that red regions indicate high attention to the model. The closer the color is to red, the higher the attention the model is paying to that region. Conversely, blue indicates low attention. P is the softmax score for that class category. In frames with high redundancy, our FAR focuses on temporal features compared to MVD.

Figure 6 shows the visualization results for four class categories where there is high redundancy between adjacent frames. In the ’Spilling something next to something’ class, our FAR compared to the MVD shows that the heatmap is focused on the liquid. This indicates that FAR better understands the key temporal aspect of the action, which is the movement of the liquid. In the ’Twisting something’ class, unlike the MVD, the FAR focuses on the hand where the action is occurring. Similarly, in the ’Stuffing something into something’ and ’Putting a number of something onto something’ classes, MVD fails to focus on the area where the action is taking place, resulting in heatmaps in the background or irrelevant regions. In contrast, FAR focuses on regions where actions occur, even in cases of high redundancy between frames These visualizations show that FAR can correctly classify actions even when there is high redundancy between frames in Something-Something V2.

**Figure 7:**
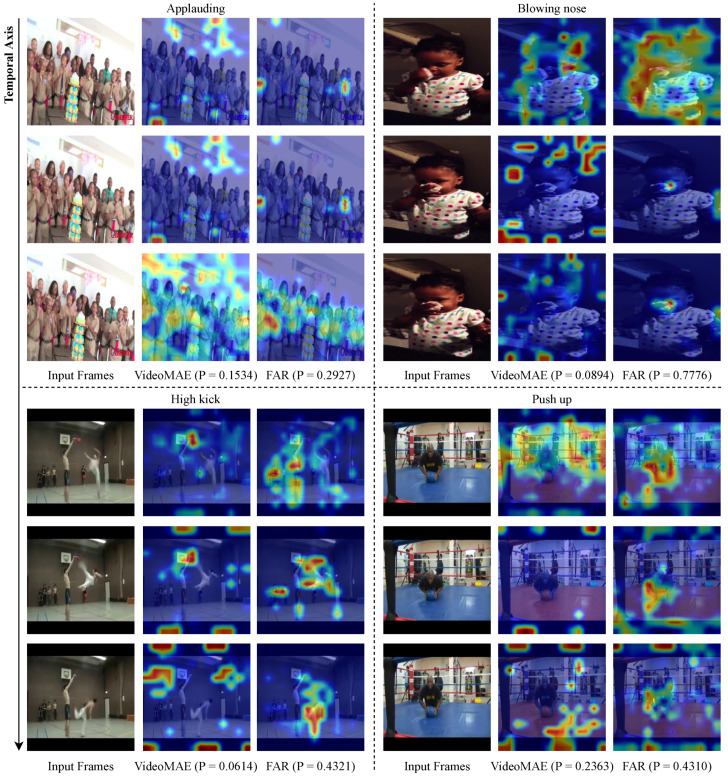
Qualitative comparison of VideoMAE and FAR in Kinetics-400. Visualized with GradCAM, the heatmap shows that red regions indicate high attention to the model. The closer the color is to red, the higher the attention the model is paying to that region. Conversely, blue indicates low attention. P is the softmax score for that class category. In frames with high redundancy, our FAR focuses on temporal features compared to MVD.

Figure 7 shows the qualitative comparison between VideoMAE and FAR in the Kinetics-400 dataset. In the ’Applauding’ class, FAR precisely focuses on the clapping hands, which are important for action recognition, whereas VideoMAE exhibits more dispersed attention, leading to a lower softmax score. For ’Blowing Nose’, FAR effectively captures the arm’s movement towards the face, which defines the action, while VideoMAE’s attention is diffused, missing key details. In the ’High Kick’ class, FAR distinctly captures the motion of the kicking leg, whereas VideoMAE fails to prioritize this key movement effectively. In the ’Push Up’ class, VideoMAE’s attention is scattered across the background, while FAR accurately concentrates on the person performing the action, resulting in a higher softmax score. These visualizations show that FAR can correctly classify actions even when there is high redundancy between frames in Kinetics-400.

FAR’s focus on temporally significant regions of the video leads to better classification results, and it is able to capture temporal information from our highly redundant frames and classify the video with higher accuracy than MVD and VideoMAE.

## 5. Conclusions

In this paper, we proposed FAR that mitigates temporal redundancy [18] for effective spatio-temporal feature modeling of video. By investigating different types of attention in the video transformer framework, we showed that HSDA can balance between temporal and spatial features. Furthermore, we use token–group interaction to overcome the problems caused by HSDA and confirm that the group-averaged classifier can effectively use the features obtained by the encoder. FAR, which can use robust weights pre-trained by masked autoencoders, such as VideoMAE [19] and MVD [9], achieves high performance compared to previous methods on the Something-Something V2 [21] and Kinetics-400 [22] datasets. These results provide an important contribution to improving model performance in video classification and recognition, and we believe it is worthwhile to further explore how to better fine-tune the pre-trained weights in future work.

### Limitations and Future Work

Our proposed FAR mitigates temporal redundancy, but it has limitations. Although we demonstrate that FAR is effective in eight experiments using VideoMAE [19] and MVD [9], which are among several recently proposed masked autoencoder pre-training methods, we cannot guarantee that FAR will be effective for other methods such as ST-MAE [20] or OmniMAE [53]. Our inability to demonstrate the generality of FAR across different pre-training methods remains a limitation of our approach, as we were unable to evaluate them due to a lack of GPU resources. Furthermore, although the proposed method successfully addresses the problem of temporal redundancy, the performance improvement in highly dynamic scenarios is uncertain, suggesting that further enhancements are needed in such cases.

Future work will explore improving the generalizability of the FAR model by validating its effectiveness across a broader range of pre-training methods and expanding its applicability to other forms of video understanding tasks, such as multi-person interactions or event detection. Potential research paths also include refining attention patterns to improve performance in highly dynamic or complex video scenarios, where temporal relationships are more challenging to capture. Moreover, extending FAR to incorporate other complementary modalities, such as audio, could provide richer spatiotemporal features, leading to more robust action recognition capabilities.

## Figures and Tables

**Table 1:** Fine-tuning setting on FAR.

	FAR (VideoMAE)		FAR (MVD)	
**Config**	**Sth-Sth V2**	**Kinetics-400**	**Sth-Sth V2**	**Kinetics-400**
optimizer	AdamW		AdamW	
learning rate	$1\times 10^{-3}$ (S), $5\times 10^{-4}$ (B)	$1\times 10^{-3}$	$1\times 10^{-3}$ (S), $5\times 10^{-4}$ (B)	$1\times 10^{-3}$
lr schedule	cosine decay		cosine decay	
weight decay	0.05		0.05	
momentum	$\beta_{1},\beta_{2}$ = 0.9, 0.999		$\beta_{1},\beta_{2}$ = 0.9, 0.999	
batch size	384	384 (S), 512 (B)	384	
accumulation step	8 (S), 16 (B)	6 (S), 16 (B)	6 (S), 4 (B)	6 (S), 16 (B)
warmup epochs	5		5	
training epochs	40 (S), 30 (B)	150 (S), 100 (B)	40 (S), 30 (B)	150 (S), 75 (B)
drop path [46]	0.1		0.1	
layer-wise lr decay [39]	0.7 (S), 0.75 (B)	0.75	0.7 (S), 0.75 (B)	0.75
repeated augmentation	2	2 (S), 1 (B)	2	
flip augmentation	no	yes	no	yes
RandAug [47]	(9, 0.5)		(9, 0.5)	
label smoothing [48]	0.1		0.1	
mixup [49]	0.8		0.8	
cutmix [50]	1.0		1.0	

**Table 2:** Comparisons of different attention patterns.

Type	Top-1 (%)	Top-5 (%)	FLOPS (G)
Global	70.7	92.8	56.9
Sparse-Dense	70.0	92.5	45.6
Temporal	47.5	76.5	34.4
Head-Split Temporal	68.4	91.6	45.7
Head-Split Sparse-Dense	70.6	92.9	51.3

**Table 3:** Comparisons of token–group interaction.

Type	Top-1 (%)	Top-5 (%)	FLOPS (G)	Param (M)
w/o Bottleneck	70.6	92.9	51.3	22.0
Bottleneck w Group	70.9	93.1	53.5	23.4
Bottleneck w/o Group	71.0	93.0	57.4	25.9

**Table 4:** Comparisons of classifier types.

Type	Top-1 (%)	Top-5 (%)	FLOPS (G)	Param (M)
Global-Mean	70.9	93.1	53.5	23.4
Group-Mean	71.1	92.9	53.5	23.4

**Table 5:** Comparisons of head-split ratios.

Ratio (Global:Sparse-Dense)	Top-1 (%)	Top-5 (%)	FLOPS (G)
1:1	71.1	92.9	53.5
2:1	70.7	92.9	55.4
1:2	70.7	93.0	51.6

**Table 6:** Comparisons of token–group interaction position.

Token–Group Interaction Position	Top-1 (%)	Top-5 (%)	FLOPS (G)
Before Attention	71.1	92.9	53.5
After Attention	70.8	92.9	53.5
After MLP	70.7	92.4	53.5

**Table 7:** Comparisons with the other methods on Something-Something V2. Our implementation and FAR use the mean of two random seed results.

Method	Extra Data	Backbone	Flops (G)	Top-1 (%)	Top-5 (%)
supervised					
SlowFast [14]	K400	ResNet101	106 × 3	63.1	87.6
MViTv1 [17]	-	MViTv1-B	455 × 3	67.7	90.9
MViTv2 [51]	K400	MViTv2-B	225 × 3	70.5	92.7
TimeSformer [15]	IN-21K	ViT-B	196 × 3	59.5	N/A
ViViT FE [11]	IN-21K	ViT-L	995 × 12	65.9	89.9
Video Swin [10]	IN-21K + K400	Swin-B	321 × 3	69.6	92.7
self-supervised					
VIMPAC [41]	HowTo100M	ViT-L	N/A × 30	68.1	N/A
BEVT [52]	IN-1K + K400	Swin-B	321 × 3	70.6	N/A
MaskFeat [44]	K400	MViT-L	2828 × 3	74.4	94.6
ST-MAE [20]	K400	ViT-L	598 × 3	72.1	93.9
OmniMAE [53]	IN-1K	ViT-B	180 × 6	69.5	N/A
	IN-1K + K400	ViT-B	180 × 6	69.0	N/A
our implementation					
VideoMAE [19]	-	ViT-S	57 × 6	66.7	90.3
	-	ViT-B	180 × 6	70.2	92.2
MVD [9]	IN-1K + K400	ViT-S	57 × 6	70.7	92.8
	IN-1K + K400	ViT-B	180 × 6	73.2	94.0
FAR (VideoMAE)	-	ViT-S	53 × 6	66.9	90.4
	-	ViT-B	176 × 6	70.4	92.1
FAR (MVD)	IN-1K + K400	ViT-S	53 × 6	71.1	92.9
	IN-1K + K400	ViT-B	176 × 6	73.3	93.9

**Table 8:** Comparisons with the other methods on Kinetics-400. Our implementation and FAR use the mean of two random seed results.

Method	Extra Data	Backbone	Flops (G)	Top-1 (%)	Top-5 (%)
supervised					
SlowFast [14]	-	R101+NL	234 × 30	79.8	93.9
MViTv1 [17]	-	MViTv1-B	170 × 5	80.2	94.4
TimeSformer [15]	IN-21K	ViT-L	2380 × 3	80.7	94.7
ViViT FE [11]	JFT-300M	ViT-L	3980 × 3	83.5	94.3
Video Swin [10]	IN-1K	Swin-B	282 × 12	80.6	94.6
self-supervised					
VIMPAC [41]	HowTo100M	ViT-L	N/A × 30	77.4	N/A
BEVT [52]	IN-1K	Swin-B	282 × 12	81.1	N/A
MaskFeat [44]	-	MViT-S	71 × 10	82.2	95.1
ST-MAE [20]	-	ViT-B	180 × 21	81.3	94.9
OmniMAE [53]	IN-1K	ViT-B	180 × 15	80.8	N/A
our implementation					
VideoMAE [19]	-	ViT-S	57 × 15	79.0	93.9
	-	ViT-B	180 × 15	80.0	94.5
MVD [9]	IN-1K	ViT-S	57 × 15	80.9	94.8
	IN-1K	ViT-B	180 × 15	83.4	95.8
FAR (VideoMAE)	-	ViT-S	53 × 15	79.4	94.0
	-	ViT-B	176 × 15	80.4	94.5
FAR (MVD)	IN-1K	ViT-S	53 × 15	81.1	94.9
	IN-1K	ViT-B	176 × 15	83.6	95.7

## Data Availability

The Something-Something V2 dataset can be downloaded from https://developer.qualcomm.com/software/ai-datasets/something-something (accessed on 2 March 2024). The Kinetics-400 dataset can be downloaded from https://github.com/cvdfoundation/kinetics-dataset (accessed on 10 June 2024).
